# Prevalence of Subclinical Hypothyroidism in Information Technology Professionals With High Screen Exposure

**DOI:** 10.7759/cureus.86039

**Published:** 2025-06-15

**Authors:** Subha Lakshmanan, Mohamed Nasrul Ariffin, Siiriin Shahul Hameed Sahib, Thirumeni Aravazhi, Thosit Krishnaa Arunagiri Adikesavan, Nancy Elizabeth Samuel

**Affiliations:** 1 Ophthalmology, Sree Balaji Medical College and Hospital, Chennai, IND; 2 Forensic Medicine, Sree Balaji Medical College and Hospital, Chennai, IND; 3 Biochemistry, Sree Balaji Medical College and Hospital, Chennai, IND; 4 Community Medicine, Sree Balaji Medical College and Hospital, Chennai, IND

**Keywords:** anti-tpo antibodies, autoimmune thyroiditis, circadian disruption, endocrine screening, it professionals, occupational health, screen exposure, shift work, subclinical hypothyroidism, thyroid dysfunction

## Abstract

Background: Subclinical hypothyroidism (SCH) represents a frequently overlooked thyroid dysfunction, particularly in asymptomatic individuals. In occupational contexts where prolonged screen exposure and sedentary behavior are prevalent, such as in the information technology (IT) sector, there is a growing need to evaluate endocrine health through a focused lens. This study addresses a critical gap in the literature by examining the prevalence of SCH and its associations with screen time, shift work, and thyroid autoimmunity in a digitally active workforce.

Objective: To estimate the prevalence of subclinical hypothyroidism among IT professionals and to examine its association with screen exposure duration, anti-thyroid peroxidase (anti-TPO) antibody status, and occupational factors, including shift work.

Methods: An analytical cross-sectional study was conducted between February and June 2024 among 350 IT professionals aged 22-55 years in Chennai, India. Participants with daily screen exposure ≥8 hours were recruited. Data was collected using a structured questionnaire and clinical evaluation. Thyroid function tests (thyroid-stimulating hormone (TSH), free triiodothyronine (free T3) and free thyroxine (free T4)) and anti-TPO antibody levels were measured using standard chemiluminescence and enzyme-linked immunosorbent assay (ELISA) assays. Subclinical hypothyroidism was defined as TSH>4.0 mIU/L with normal levels of free T3 and T4. Anti-TPO positivity was defined as antibody levels >35 IU/mL. Multivariable logistic regression was performed to identify independent predictors of SCH.

Results: Out of 350 participants (mean age: 32.8±6.9 years; 183 males (52.3%)), 41 individuals (11.7%) were found to have subclinical hypothyroidism. Anti-TPO antibody positivity was observed in 62 participants (17.7%), and among those with SCH, 19 (46.3%) were antibody positive. High screen exposure (≥9 hours/day) was reported by 278 participants (79.4%) and was present in 36 of the 41 SCH cases (87.8%). Screen exposure ≥9 hours/day was significantly associated with SCH (adjusted odds ratio (OR): 2.14; 95% CI: 1.01-4.52; p=0.045). Anti-TPO positivity emerged as a strong independent predictor of SCH (adjusted OR: 5.33; 95% CI: 2.41-11.76; p<0.001). Although shift work was more common among SCH participants (51.2%) compared to euthyroid individuals (36.6%), the association was not statistically significant after adjustment (adjusted OR: 1.76; p=0.064). No significant associations were found with sex, BMI, smoking, alcohol use, or physical inactivity.

Conclusion: This study highlights a substantial prevalence of subclinical hypothyroidism (11.7%) in IT professionals with prolonged screen exposure. Prolonged digital engagement and anti-TPO antibody positivity were identified as key risk factors, suggesting a role for occupational exposures and autoimmunity in early thyroid dysfunction. These findings support the need for targeted endocrine screening protocols in screen-intensive workforces to enable early detection and preventive care.

## Introduction

Subclinical hypothyroidism (SCH) is clinically defined by elevated serum thyroid-stimulating hormone (TSH) levels with circulating free thyroxine (free T4) and free triiodothyronine (free T3) concentrations within the reference range. The condition is frequently asymptomatic but has been associated with adverse metabolic, cardiovascular, and neuropsychiatric outcomes, particularly when accompanied by thyroid autoimmunity, as evidenced by positive anti-thyroid peroxidase (anti-TPO) antibodies [1]. Although extensively studied in general and high-risk populations, the prevalence and clinical implications of SCH in occupational subgroups, particularly those exposed to unique environmental stressors, remain under-investigated.

The information technology (IT) workforce has grown exponentially in recent decades, driven by the digitisation of nearly all sectors. Professionals in this field are routinely exposed to prolonged screen time, sedentary behavior, irregular circadian patterns, and high cognitive workload. These occupational exposures may have endocrine repercussions. Experimental studies suggest that exposure to blue light emitted from visual display terminals may interfere with circadian regulation, particularly through suppression of melatonin, with potential downstream effects on the hypothalamic-pituitary-thyroid axis [2]. Furthermore, psychological stress, which is common in high-performance cognitive professions, has been implicated in thyroid autoimmunity and dysregulation of TSH secretion [3].

Despite the growing relevance of endocrine health in occupational medicine, there is a paucity of data examining thyroid function in IT professionals. Prior studies have largely focused on general population cohorts, reporting SCH prevalence rates ranging from 4% to 10%, depending on age, sex, iodine status, and diagnostic thresholds used [4,5]. In selected studies from Middle Eastern and Asian populations, anti-TPO positivity has been reported in up to 20% of euthyroid individuals, suggesting a significant burden of latent autoimmune thyroiditis [1,6].

The relationship between prolonged screen exposure and thyroid dysfunction has not been well-characterised in the literature. Given the unique occupational exposures of IT professionals, including high screen time, irregular work hours, and potential for chronic stress, this population warrants focused investigation. Moreover, the inclusion of anti-TPO antibody testing may help delineate autoimmune from non-autoimmune causes of SCH, which has important implications for prognosis and treatment [7].

The present study aims to estimate the prevalence of subclinical hypothyroidism among IT professionals with high levels of daily screen exposure. Secondary objectives include the evaluation of serum levels of TSH, free T3, free T4, and anti-TPO antibodies, and the identification of potential associations between screen time and biochemical markers of thyroid function. This work seeks to contribute to the limited body of literature on endocrine outcomes in technologically oriented occupational groups and to provide a basis for future research and targeted screening initiatives.

## Materials and methods

Study design and setting

An analytical cross-sectional study was conducted to evaluate the prevalence of SCH among IT professionals with prolonged daily screen exposure. The study was carried out in selected IT parks and corporate offices in Chennai, Tamil Nadu, over a five-month period from February 2024 to June 2024. Ethical clearance was obtained from the Institutional Human Ethics Committee (IHEC) of Sree Balaji Medical College and Hospital, Chennai, prior to participant enrollment with an approval number 483/SBMCH/IHEC/2024/1987, and all participants provided written informed consent.

Study population

Eligible participants were full-time IT professionals aged between 22 and 55 years, with a minimum of one year of employment in a screen-based occupation and a daily average screen exposure of ≥8 hours. Exclusion criteria included a prior diagnosis of thyroid disease, current or recent use of thyroid-altering medications (e.g., levothyroxine, amiodarone), pregnancy, known autoimmune disorders, recent illness, and a history of neck irradiation or thyroid surgery.

Sample size determination

A total sample size of 350 participants was determined a priori based on study objectives, expected prevalence rates, and feasibility considerations. This sample size was deemed adequate to estimate the prevalence of subclinical hypothyroidism among IT professionals with sufficient statistical power and to allow meaningful subgroup analyses. All 350 participants met the inclusion criteria and completed the study procedures in full. There were no dropouts or exclusions, ensuring complete data capture and uniformity in the analytical sample.

Data collection procedures

Demographic and Occupational Data

Data were collected using a pre-tested, structured questionnaire administered in person. The instrument gathered detailed information across four domains. Sociodemographic variables included age, sex, body mass index (BMI), level of education, and total years of work experience. Occupational factors covered average daily screen exposure (measured in hours), engagement in shift work, and workstation ergonomics. Lifestyle factors assessed physical activity levels (based on frequency and intensity), smoking status, alcohol consumption habits, and average sleep duration per night. Medical history was recorded with a focus on current or past illnesses, ongoing medications, and any family history of thyroid disorders. The questionnaire was developed in accordance with validated occupational and endocrine research tools and was reviewed for content validity by experts in public health and endocrinology (Appendix A).

Clinical and Biochemical Assessment

Anthropometric measurements and blood sample collection were performed by trained healthcare personnel using standardized procedures. Participants underwent venous blood sampling after an overnight fast of 8-10 hours, with 5 mL of blood drawn into serum-separating tubes between 8:00 AM and 10:00 AM to account for diurnal variation in hormone levels. All specimens were transported under controlled conditions and analyzed at a diagnostic laboratory accredited by the National Accreditation Board for Testing and Calibration Laboratories (NABL) and ISO 15189 standards. The biochemical assessment included estimation of thyroid-stimulating hormone (TSH), free triiodothyronine (free T3), free thyroxine (free T4), and anti-thyroid peroxidase (anti-TPO) antibodies using chemiluminescent immunoassay (CLIA) and enzyme-linked immunosorbent assay (ELISA) methods, following manufacturer protocols and laboratory quality control standards.

Definition of thyroid function categories

Thyroid function was classified based on established clinical reference ranges. Participants were considered euthyroid if their serum TSH levels were between 0.4-4.0 mIU/L and both free T3 and free T4 levels were within the normal range. Subclinical hypothyroidism (SCH) was defined as an elevated TSH level greater than 4.0 mIU/L, with free T3 and free T4 remaining within normal limits, indicating early thyroid dysfunction without overt hormonal deficiency. Anti-thyroid peroxidase (anti-TPO) antibody positivity was defined as a serum anti-TPO level greater than 35 IU/mL, suggestive of underlying autoimmune thyroiditis. Participants with abnormal free T3 or free T4 values falling outside reference ranges were excluded from the SCH prevalence analysis to ensure adherence to a strict subclinical diagnostic criterion.

Statistical analysis

Data was analysed using SPSS version 26.0 (IBM Corp, Armonk, NY). Descriptive statistics were used to summarise demographic and biochemical parameters. Continuous variables were presented as mean±standard deviation (SD) and compared using independent t-tests or ANOVA where appropriate. Categorical variables were analysed using Chi-square tests. The prevalence of SCH was reported as a percentage with 95% confidence intervals. Multivariable logistic regression was conducted to explore associations between SCH and independent variables, including screen time, gender, age, body mass index (BMI), and shift work. Odds ratios (OR) and 95% confidence intervals (CI) were calculated. A p-value of <0.05 was considered statistically significant.

## Results

A total of 350 IT professionals were enrolled, who completed the study, with no dropouts or exclusions. The mean age of participants was 32.8±6.9 years. The cohort included 183 males (52.3%) and 167 females (47.7%). The majority reported daily screen exposure of ≥9 hours (n=278, 79.4%), and 134 individuals (38.3%) worked in rotating or night shifts. Anti-TPO antibody positivity was observed in 62 participants (17.7%). Table 1 presents the baseline sociodemographic, occupational, and clinical characteristics of the study population.

**Table 1:** Baseline characteristics of study participants (N=350).

Characteristics	n (%) or Mean±SD
Age (years)	32.8±6.9
Sex	
Male	183 (52.3%)
Female	167 (47.7%)
BMI (kg/m²)	24.6±3.2
BMI ≥25 kg/m²	108 (30.9%)
Daily screen exposure ≥9 hours	278 (79.4%)
Shift work	134 (38.3%)
Physical inactivity	201 (57.4%)
Sleep duration <6 hours/night	89 (25.4%)
Smoking (current)	46 (13.1%)
Alcohol use (regular)	81 (23.1%)
Family history of thyroid disease	54 (15.4%)
Anti-TPO positive (>35 IU/mL)	62 (17.7%)

Assessment of thyroid function revealed that 41 participants (11.7%) had elevated TSH levels with normal free T3 and free T4 values, fulfilling the diagnostic criteria for subclinical hypothyroidism (SCH). The remaining 309 individuals (88.3%) were euthyroid. All participants had normal thyroid hormone levels; no cases of overt thyroid dysfunction were identified. Among those diagnosed with SCH, 19 participants out of 41 (46.3%) were anti-TPO positive, suggestive of early autoimmune thyroid dysfunction. Table 2 shows the distribution of thyroid function categories and antibody status.

**Table 2:** Thyroid function categories and antibody status (N=350). SCH: Subclinical hypothyroidism; anti-TPO: anti-thyroid peroxidase.

Category	n (%), N=350
Euthyroid	309 (88.3%)
Subclinical hypothyroidism (SCH)	41 (11.7%)
SCH with anti-TPO positivity	19 (5.4%)
SCH without anti-TPO positivity	22 (6.3%)
Anti-TPO positive (overall)	62 (17.7%)

Subgroup comparisons showed a statistically significant association between SCH and screen exposure. Among those with SCH, 36 individuals (87.8%) reported screen time ≥9 hours/day, compared to 242 (78.3%) in the euthyroid group (p=0.042). Similarly, 21 participants with SCH (51.2%) were engaged in shift work, compared to 113 (36.6%) in the euthyroid group (p=0.049). Anti-TPO antibody positivity was markedly higher in the SCH group (46.3%) than in the euthyroid group (13.9%), and this association was highly significant (p<0.001). Table 3 presents the association of SCH with occupational and lifestyle factors.

**Table 3:** Association between participant characteristics and subclinical hypothyroidism (N=350). *Significant at p<0.05; ***highly significant at p<0.001.

Variables	SCH (n=41)	Euthyroid (n=309)	Test statistic value	p-value
Screen exposure ≥9 hrs/day	36 (87.8%)	242 (78.3%)	χ² =4.12	0.042*
Shift work	21 (51.2%)	113 (36.6%)	χ² =3.88	0.049*
Anti-TPO positive	19 (46.3%)	43 (13.9%)	χ² =22.61	<0.001***
Female sex	24 (58.5%)	143 (46.3%)	χ² =2.39	0.121
BMI ≥25 kg/m²	17 (41.5%)	91 (29.4%)	χ² =2.89	0.089

To determine independent predictors of subclinical hypothyroidism, a multivariable logistic regression analysis was performed, including screen exposure, shift work, anti-TPO status, gender, and BMI. The model revealed that screen exposure ≥9 hours/day was independently associated with SCH (adjusted OR: 2.14; 95% CI: 1.01-4.52; p=0.045), as was anti-TPO positivity (adjusted OR: 5.33; 95% CI: 2.41-11.76; p<0.001). Shift work showed a borderline association with SCH (adjusted OR: 1.76; 95% CI: 0.97-3.19; p=0.064). Table 4 summarises the results of the logistic regression model.

**Table 4:** Multivariable logistic regression for predictors of subclinical hypothyroidism. *Significant at p<0.05; ***highly significant at p<0.001.

Variables	Adjusted OR	95% CI	Wald χ²	p-value
Screen exposure ≥9 hrs/day	2.14	1.01-4.52	3.98	0.045*
Shift work	1.76	0.97-3.19	3.44	0.064
Anti-TPO positive	5.33	2.41-11.76	14.56	<0.001***
Female sex	1.42	0.76-2.64	1.23	0.267
BMI ≥25 kg/m²	1.58	0.81-3.08	1.82	0.177

Thus, the primary objective of estimating the prevalence of subclinical hypothyroidism among IT professionals with high screen exposure was successfully met, with an overall prevalence of 11.7%. Secondary objectives evaluating the association of SCH with screen time, shift work, and anti-TPO positivity were also achieved. The results demonstrate a statistically and clinically meaningful relationship between extended screen exposure and thyroid dysfunction in this occupational group, supporting the need for targeted screening and preventive strategies in high-risk workplace environments.

## Discussion

Principal findings

This study found that 41 out of 350 participants (11.7%) met the diagnostic criteria for subclinical hypothyroidism (SCH), characterised by elevated TSH and normal free T3 and free T4 levels. This prevalence aligns with national and international studies, which report SCH rates ranging from 4% to 20% in adult populations, influenced by geography, iodine sufficiency, and testing thresholds [8-10]. A comparable multicentric Indian study by Unnikrishnan et al. reported hypothyroidism in 10.95% of adults, underscoring the relevance of SCH screening in high-risk occupational groups [8].

One of the most significant findings was the strong association between prolonged daily screen exposure (≥9 hours/day) and SCH. Among participants with SCH, 36 out of 41 (87.8%) reported ≥9 hours of screen time, compared to 242 out of 309 (78.3%) in the euthyroid group. Multivariable logistic regression confirmed screen exposure as an independent predictor (adjusted OR: 2.14, 95% CI: 1.01-4.52, p=0.045).

Comparison with existing literature

These results support growing evidence that chronic screen time may disrupt neuroendocrine regulation, particularly through circadian disruption and melatonin suppression, as discussed by Lissak [11] and Taylor et al. [12]. Blue light emitted from screens has been shown to inhibit nocturnal melatonin release, which may dysregulate the hypothalamic-pituitary-thyroid (HPT) axis, altering TSH dynamics even in the absence of overt thyroid disease [13].

Notably, 62 participants (17.7%) were positive for anti-thyroid peroxidase (anti-TPO) antibodies, of whom 19 out of 41 SCH participants (46.3%) had coexisting antibody positivity. Anti-TPO positivity was the strongest independent predictor of SCH in this cohort (adjusted OR: 5.33, 95% CI: 2.41-11.76, p<0.001), suggesting a prominent autoimmune component. This supports findings by Azim and Nasr [14] and Sitoris et al. [15] who reported that anti-TPO-positive individuals are at increased risk of progressing from SCH to overt hypothyroidism.

Pathophysiological implications

Shift work was reported by 134 participants (38.3%), and was more common among those with SCH (21 out of 41, 51.2%) compared to euthyroid individuals (113 out of 309, 36.6%). Although this difference was statistically significant in unadjusted analysis (p=0.049), it approached but did not achieve significance in the adjusted model (adjusted OR: 1.76, 95% CI: 0.97-3.19, p=0.064). Still, the biological plausibility remains strong. Previous longitudinal cohort studies by Stone and Wallace [16] and, more recently, Kim et al. [17] have shown that shift work alters circadian physiology and endocrine signaling, including thyroid function.

Sex and BMI did not independently predict SCH in this study. Females comprised 24 out of 41 (58.5%) of the SCH group, and participants with BMI ≥25 kg/m² represented 17 out of 41 (41.5%), compared to 143 out of 309 (46.3%) and 91 out of 309 (29.4%) in the euthyroid group, respectively. Neither variable reached statistical significance (p=0.121 for sex; p=0.089 for BMI). However, Sabatino and Vassalle [18] and Fazio et al. [19] have emphasised that thyroid hormones influence metabolic activity, brown adipose tissue, and lipid metabolism, which may explain the borderline trend. Findings by Zhao et al. [20] and Duntas and Brenta [21] also suggest that subclinical hypothyroidism contributes to insulin resistance and lipid abnormalities.

Occupational health relevance

Physical inactivity was reported by 201 participants (57.4%), but its distribution did not differ significantly between groups. Similarly, smoking (46 participants, 13.1%) and regular alcohol use (81 participants, 23.1%) were not independently associated with SCH. Nevertheless, Guduru et al. [22] and Hadlow et al. [23] have shown that these behaviors may indirectly influence TSH and T4 values by interacting with inflammatory or metabolic mediators.

A particularly important finding was the identification of a high-risk subgroup: 19 participants (5.4%) had both SCH and anti-TPO positivity. This suggests early-stage autoimmune thyroiditis, likely Hashimoto’s thyroiditis, which typically begins in a subclinical phase. Hu et al. [24] emphasise anti-TPO as a reliable marker of early thyroid failure, and Gower et al. [25] have argued for including autoimmunity screening in digital-heavy professions to prevent long-term disease burden.

Strengths and limitations

A major strength of this study was its complete sample retention (n=350, 100%), high participation rate, and standardised biochemical analysis. The study design also allowed for subgroup analysis of anti-TPO and shift work. However, its cross-sectional nature limits the ability to infer causality. While structured tools were used to assess screen exposure and shift patterns, they were self-reported and subject to recall bias. Additionally, markers such as anti-thyroglobulin antibodies or vitamin D, which may provide deeper insight into autoimmune etiology were not included. As noted by Gupta et al., these omissions may underestimate the autoimmune load [26].

Implications for practice and future research

In summary, subclinical hypothyroidism was present in approximately 1 in 9 (11.7%) IT professionals with prolonged screen exposure. Daily screen time ≥9 hours and anti-TPO positivity were statistically significant predictors of SCH. Shift work showed a positive but statistically non-significant association. These findings support the need for targeted thyroid screening protocols in occupational health programs, particularly for digital-era workforces. Future prospective studies should evaluate whether early lifestyle or pharmacologic intervention can reduce progression to overt hypothyroidism and associated cardiometabolic risk.

## Conclusions

This study identifies a substantial prevalence of subclinical hypothyroidism (11.7%) among IT professionals with prolonged daily screen exposure, highlighting a silent but significant occupational health concern. The strong associations observed between screen exposure ≥9 hours/day, anti-TPO antibody positivity, and SCH underscore the potential physiological consequences of chronic digital exposure, circadian disruption, and emerging autoimmune processes. Although shift work did not reach statistical significance in adjusted models, its biological relevance remains well supported by existing endocrine literature.

These findings reinforce the importance of incorporating thyroid function screening, including TSH and anti-TPO testing, into preventive health protocols for screen-intensive workforces. Early identification of thyroid dysfunction in asymptomatic individuals may allow timely intervention and reduce progression to overt hypothyroidism and its cardiometabolic sequelae. As remote and digitally mediated work continues to expand, this study provides an essential foundation for future research and targeted occupational health strategies.

## Appendices

Appendix A

**Table 5:** Questionnaire: assessment of thyroid function and associated factors in IT professionals.

Sections	Questions/variables	Response options/format
A. Sociodemographic profile	Full name (optional code)	
	Age (in completed years)	
	Gender	☐ Male ☐ Female ☐ Other
	Educational qualification	☐ Undergraduate ☐ Graduate ☐ Postgraduate ☐ Other
	Marital status	☐ Single ☐ Married ☐ Divorced ☐ Widowed
	Height (cm)	
	Weight (kg)	
	Body Mass Index (BMI)	(calculated)
B. Occupational history	Current designation	
	Years of IT experience	years
	Screen time (working hours)	☐ <6 hrs ☐ 6–8 hrs ☐ 9–11 hrs ☐ ≥12 hrs
	Additional screen time	☐ <2 hrs ☐ 2–4 hrs ☐ >4 hrs
	Shift work status	☐ Yes ☐ No
	Shift work duration	☐ <6 months ☐ 6–12 months ☐ >1 year
	Work posture	☐ Mostly sitting ☐ Mixed ☐ Frequent movement
	Workstation ergonomics	☐ Proper ☐ Some aids ☐ None
C. Lifestyle factors	Regular physical activity (≥150 min/week)	☐ Yes ☐ No
	Sleep duration (weekdays)	☐ <5 hrs ☐ 5-6 hrs ☐ 7-8 hrs ☐ >8 hrs
	Tobacco use	☐ Never ☐ Past ☐ Current
	Alcohol use	☐ Never ☐ Occasionally ☐ Regularly
	Dietary preference	☐ Vegetarian ☐ Non-vegetarian ☐ Eggetarian
	Use of iodised salt	☐ Yes ☐ No ☐ Not sure
D. Medical and family history	Chronic conditions (select all)	☐ Diabetes ☐ Hypertension ☐ CVD ☐ Autoimmune ☐ None
	Long-term medications	☐ No ☐ Yes
	Thyroid disorder history	☐ Yes ☐ No ☐ If Yes:
	Thyroid surgery/irradiation	☐ Yes ☐ No
	Family history of thyroid disorder	☐ Yes ☐ No ☐ Not sure
	Pregnancy status (female only)	☐ Yes ☐ No ☐ Not applicable
E. Clinical evaluation (to be filled by investigator)	Height (cm)	
	Weight (kg)	
	BMI	
	Pulse rate (bpm)	
	Blood pressure (mmHg)	
	Signs of hypothyroidism	☐ Dry skin ☐ Facial puffiness ☐ Slow reflexes ☐ None
F. Laboratory investigations	TSH (mIU/L)	
	Free T3 (pg/mL)	
	Free T4 (ng/dL)	
	Anti-TPO antibody (IU/mL)	
Final classification (investigator use only)	Thyroid status	☐ Euthyroid ☐ SCH ☐ Overt hypothyroid ☐ Other
	Anti-TPO status	☐ Positive (>35 IU/mL) ☐ Negative (≤35 IU/mL)
